# A Wireless 2-Channel Layered EMG/NIRS Sensor System for Local Muscular Activity Evaluation

**DOI:** 10.3390/s23208394

**Published:** 2023-10-11

**Authors:** Akira Kimoto, Yuya Oishi, Masanao Machida

**Affiliations:** Faculty of Science and Engineering, Saga University, Saga 840-8502, Japanmmasanao@cc.saga-u.ac.jp (M.M.)

**Keywords:** electromyography, near-infrared spectroscopy, local muscular activity, layered sensor

## Abstract

A wireless 2-channel layered sensor system that enables electromyography (EMG) and near-infrared spectroscopy (NIRS) measurements at two local positions was developed. The layered sensor consists of a thin silver electrode and a photosensor consisting of a photoemitting diode (LED) or photodiode (PD). The EMG and NIRS signals were simultaneously measured using a pair of electrodes and photosensors for the LED and PD, respectively. Two local muscular activities are presented in detail using layered sensors. In the experiments, EMG and NIRS signals were measured for isometric constant and ramp contractions at each forearm using layered sensors. The results showed that local muscle activity analysis is possible using simultaneous EMG and NIRS signals at each local position.

## 1. Introduction

In sports science and physical rehabilitation, it is necessary to develop wearable sensors for monitoring local muscular activity, particularly for detecting local muscular fatigue. Many researchers have developed sensors that enable the analysis of local muscular activity and estimation of local muscular fatigue using sensing methods, such as electromyography (EMG), mechanomyography (MMG), and near-infrared spectroscopy (NIRS). EMG measures the electrical voltage generated by muscular contractions using dish electrodes pasted onto the skin surface and is widely used as a standard method for evaluating muscular activity involving muscular fatigue [1,2,3,4,5]. MMG measures the mechanical vibrations generated by muscle contraction using accelerometers, microphones, and piezoelectric elements arranged on the skin surface [6,7,8,9,10,11], while NIRS measures the variations in oxygen consumption generated by muscular contractions using a light-emitting diode (LED) and a photodiode (PD) [12,13,14,15,16]. Additionally, multimodal sensors based on combinations of EMG and MMG [17,18,19], EMG and NIRS [20,21,22,23], and EMG, MMG, and NIRS [24] have been developed for muscular activity analysis and the detailed detection of muscular fatigue. Recently, wireless, compact, and portable sensor systems with EMG and MMG sensors [25,26], EMG and NIRS [27,28] sensors, and EMG, MMG, and electrical impedance myography sensors [29] have been utilized. However, it is not sufficient to estimate muscular fatigue at multiple local positions using many developed sensors.

This study aimed to develop a wearable-type sensor for analyzing multiple local muscular activities, including the prediction of muscular fatigue, that is, detection of the anaerobic threshold (AT). A multilayered sensor was developed and simultaneous measurements of EMG, MMG, and NIRS signals were demonstrated to estimate AT [30]. Next, a wireless multi-layered sensor was developed, and its usefulness was demonstrated by analyzing the local muscular activity, including at the lateral vastus muscle, during cycling exercises [31]. Furthermore, it was found that the sensor had the potential to detect AT because AT was detected based on the ratio of Hb to EMG signals. Although it is possible to measure local muscular activity at one position using a sensor, it is difficult to measure local muscular activity at multiple positions. It is necessary to develop a sensor that allows measurements of local muscular activities at multiple positions because it is important to predict which local muscle is fatigued. Therefore, a wireless 2-channel layered sensor system that allows the simultaneous measurement of EMG and NIRS at two positions was developed based on the relationship between AT and the ratio of EMG and NIRS. One advantage of the sensor is the ease of adjusting the distance between a pair of sensors in EMG and NIRS measurements. Additionally, measuring EMG and NIRS at multiple positions is simplified using several combinations, such as one sensor with an LED and two sensors with a PD. In the experiments, EMGs and NIRSs were measured at both forearms for isometric constant contraction and isometric ramp contraction, and the usefulness of the developed sensor was evaluated.

## 2. Methods

### 2.1. Sensing System

**Figure 1:**
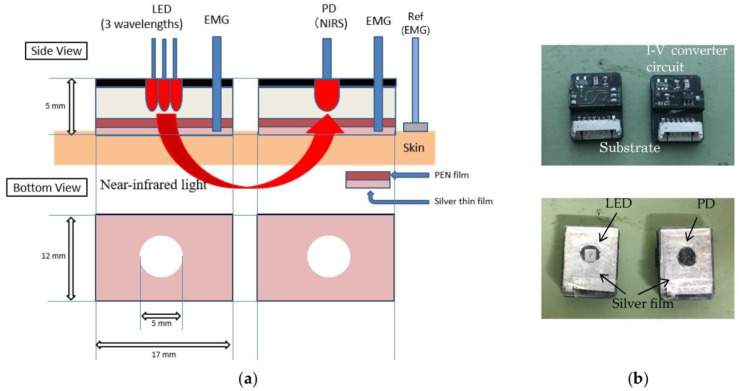
Layered sensor: (**a**) schematic diagrams of the sensor and (**b**) photographs of sensors.

Figure 1 shows a schematic diagram of the sensing method based on a pair of layered sensors with an LED or PD and photographs of the layered sensors. The sensor (12 mm × 17 mm × 5 mm) comprised a substrate with a surface-mounted LED with three types of peak wavelengths: 770 nm, 805 nm, and 870 nm (SMT770/805/870-40B 59-1, Epitex Inc., Kyoto, Japan), or a PD with spectral sensitivity between 400 nm and 1100 nm (HP601, Kodenshi Corp., Kyoto, Japan), and a thin silver film with a hole of 5 mm-diameter. The LED and PD were arranged in the holes of the silver film. A polyethylene naphthalate (PEN) film with a thickness of 12 μm was used as the insulator between the substrate and the silver film. Additionally, the circuit for the current induced by the PD is converted to a voltage arranged at the bottom of the substrate with the PD. The EMG signal was measured using a pair of silver films and a reference electrode (Ref, NE-121J, Nihon Koden Corp., Tokyo, Japan). In the NIRS signal, the voltages induced at the PD were measured by assessing which three types of lights were sequentially emitted toward the muscle by the LED. The absorbance was calculated from the voltages and changes in the concentration (Δ*C*) of oxyhemoglobin (HbO_2_). Deoxyhemoglobin (Hb) was derived using the modified Lambert–Beer law [12,14]. The EMG and NIRS signals were measured simultaneously, using a pair of sensors. Local muscular activities can be measured using 2-channel layered sensors with LED and PD.

### 2.2. Signal Processing Method

**Figure 2:**
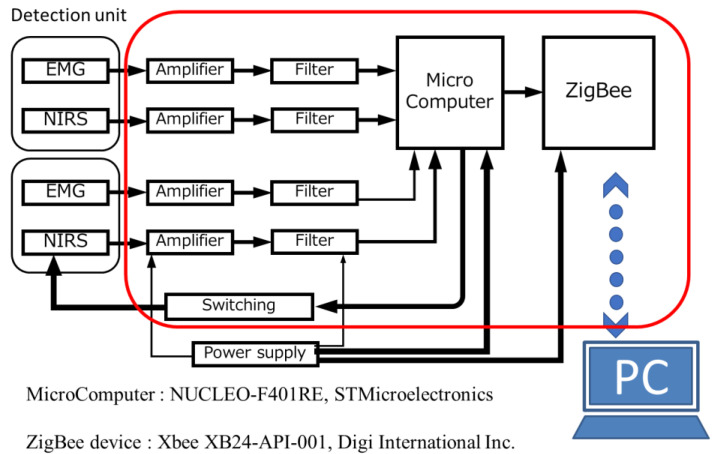
Schematic diagram of data processing circuit for EMG and NIRS.

**Figure 3:**
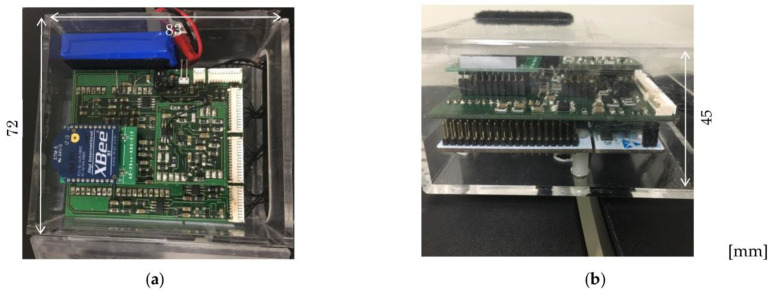
Photographs of developed signal processing circuit: (**a**) top view (**b**) side view.

Figure 2 shows a schematic diagram of the signal processing circuit for 2-channel EMG and NIRS measurements. Figure 3 shows a photograph of the developed signal-processing circuit (72 mm × 100 mm × 55 mm). Portable signal processing circuits that allow wireless 2-channel EMG and NIRS measurements were developed. The overall mass of the signal processing circuit shown in Figure 3 was approximately 240 g. The signal processing circuit primarily comprised amplification and filter circuits, and a switching circuit for emitting each LED. The EMG signals were passed through a differential amplifier (Gain:10) based on AD627 (Analog Devices Inc., Wilmington, MA, USA), for noise rejection and signal amplification. Furthermore, they were processed by two multiple feedback band-pass filters (center frequency:100 Hz, Q:0.3, Gain:12) based on AD8607 (Analog Devices Inc., USA) and a twin T notch filter based on a CR circuit. The NIRS signals were processed by two low-pass filters using AD8607 (cutoff frequency:23 Hz, Gain:8) and the notch filter. A microcomputer (Nucleo Board STM32F401RE; STMicroelectronics, Geneva, Switzerland) with a 12-bit 5-channel analog-to-digital converter was used for data acquisition and measurement control. The ZigBee module (XBee 802.15.4 S1; Digi International Inc., Hopkins, MN, USA) was used for wireless data communication between the microcomputer and PC. Two EMG and two NIRS signals were respectively acquired at equal intervals by two types of interrupt handlers with 100 μs and 40 ms [31]. Each signal of EMG and NIRS signals was sequentially measured at an interval of 400 μs (2.5 kS/s), and the average values were transmitted to the PC at an interval of 160 ms (6.25 S/s), with a serial baud rate of 38,400, respectively. Thus, the average value was obtained using 400 absolute values of voltages in EMG. The average in NIRS was obtained using 50 absolute values of voltages as data number from 351 to 400, and the error induced by the switching of LEDs was removed. The concentrations of Hb and HbO_2_ were obtained at intervals of 480 ms, as the average values of the three types of light were derived.

In the processing circuit, a voltage of 3.3 V for wireless communication and a voltage of 5.0 V for circuit operation were derived from a 7.4 V lithium battery (LI-7100SP; 1100 mAh, 42 g, S.T.L.JAPAN, Izumisano, Japan). The previous experiment demonstrated that the voltage sources remained stable with fluctuations of ±0.05% and attenuation of 0.2% over a period of 8 h.

## 3. Experiments

Two types of experiments, isometric constant contraction and isometric ramp contraction of both forearms were conducted on three healthy male volunteers in their twenties. Study participation was confirmed to be in accordance with the Declaration of Helsinki. First, the experimental and risk procedures approved by the Ethics Committee of Saga University are explained. Informed consent was obtained from all volunteers.

**Figure 4:**
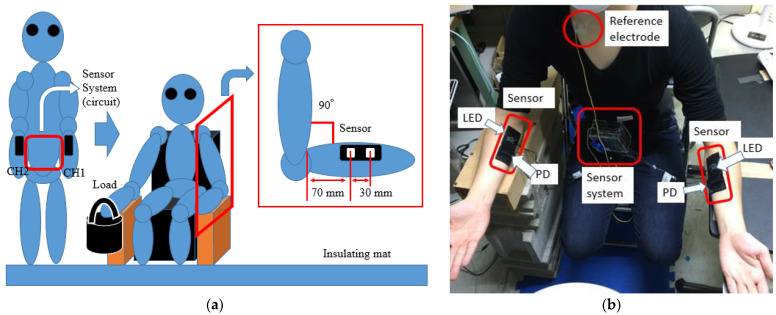
Schematic diagram of the experimental method: (**a**) Schematic diagram of experimental setup (**b**) photograph of experimental setup.

Figure 4a shows a schematic diagram of the experimental setup. Figure 4b shows a photograph of the experimental setup. The volunteers sat on a chair, and their forearms, from the elbow to the wrist, were rested on a table at a 90° angle between the upper arm and forearm. The sensors with an LED and a PD were arranged on the surface of the extensor carpi radialis longus muscle at each forearm. The sensors with an LED were pasted on the volunteer’s skin, 70 mm from the inside bend of the elbow. Sensors with a PD were also pasted in the direction of the wrist with a center-to-center distance of 30 mm between the sensors. The sensors were pasted onto a transparent conductive gel (HIT-B3M, Sekisui Kasei Co. Ltd., Osaka, Japan). The reference dish electrode was pasted onto the surface of the volunteer’s right clavicle for EMG, using a conductive paste (Elefix Z-181BE; Nihon Koden Corp., Tokyo, Japan). The sensors were covered with soft black silicone sheets to eliminate the influence of ambient light. The maximum voluntary contraction (100% MVC) force of each volunteer was measured using a myodynamometer (μTas F-1; Anima Corp., Tokyo, Japan) before the experiment was conducted. The determination of 100% MVC was conducted as follows: initially, a myodynamometer and void container (Figure 4a) were placed on the palm of the hand. Water was pumped into each container. The weight at which the hand could not be kept horizontal was used as 100% MVC. 100% MVC of each volunteer was obtained from an average of the weights at three times.

**Figure 5:**
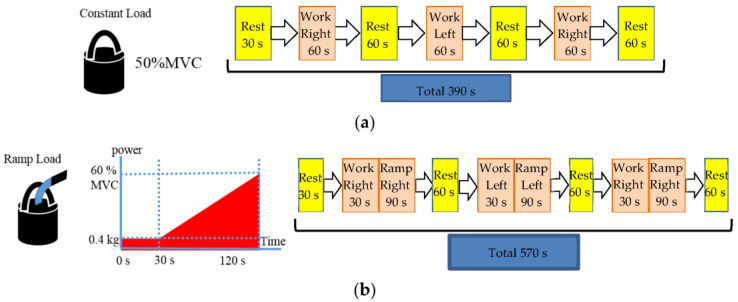
Flowchart of two types of experimental methods: (**a**) isometric constant contraction (**b**) isometric ramp contraction.

Figure 5 shows a flowchart of the isometric constant and ramp contractions in both forearms in the two types of experiments. During the isometric constant contraction experiment, both forearms were maintained in a relaxed state for 30 s (rest). Next, the work condition was introduced, which involved applying a load of 50% MVC using a container filled with water on the right palm, and this phase lasted for 60 s (work). Furthermore, another 60 s rest period was maintained, and the work phase for 60 s was alternated between the left and right palms, with each work phase lasting for 60 s and a 60 s Rest period in between. Finally, a rest of 60 s was pursued. Although the isometric ramp contraction experiment was conducted with the isometric constant contraction, the working conditions were changed. Namely, a void container was placed on the palm for 30 s, and the ramp load, which was changed by flowing water into the container at a constant rate of loading as the load reached 60% MVC at 90 s, was applied. Each experiment was conducted once per volunteer. The start of the experiment was determined by irradiating the sensor to saturate the voltage of the NIRS measurement.

## 4. Results and Discussion

**Figure 6:**
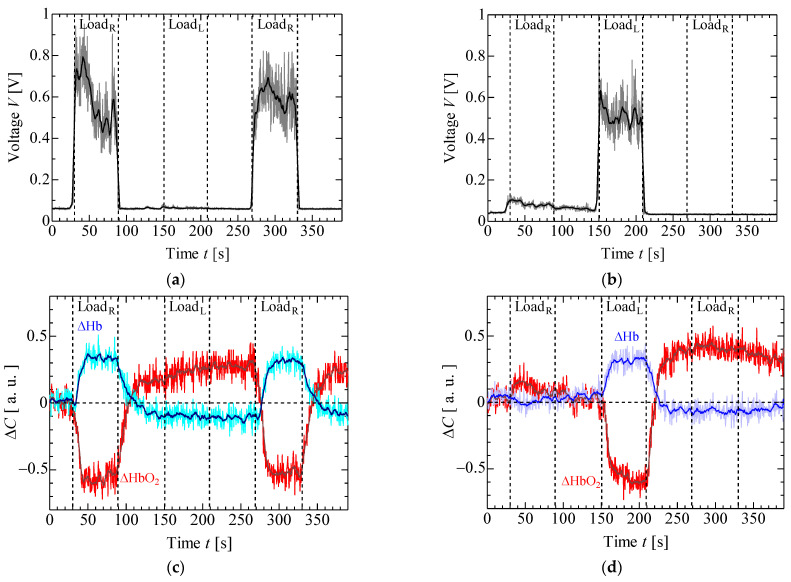
Experimental results of one volunteer in isometric constant contraction: EMG at (**a**) right forearm, (**b**) left forearm, NIRS at (**c**) right forearm and (**d**) left forearm.

**Figure 7:**
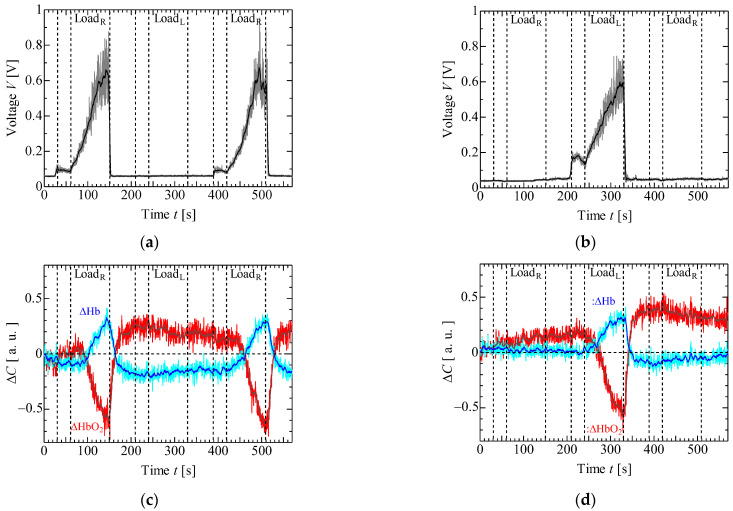
Experimental results of one volunteer in isometric ramp contraction: EMG at (**a**) right forearm, (**b**) left forearm, NIRS at (**c**) right forearm and (**d**) left forearm.

**Figure 8:**
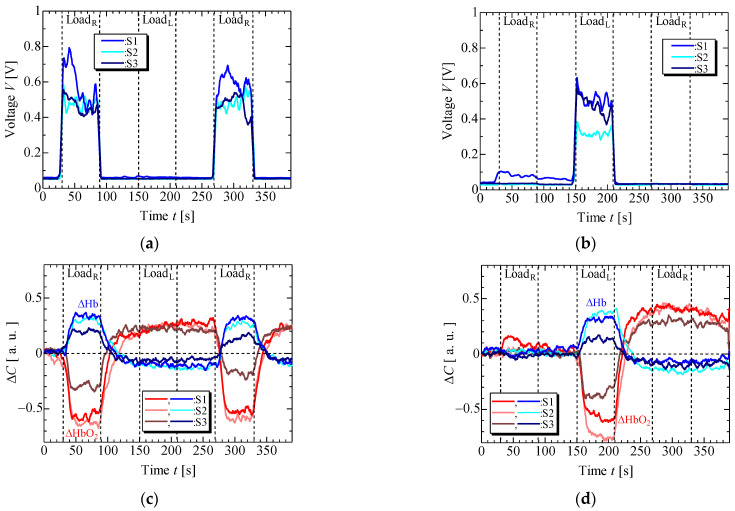
Experimental results of three volunteers in isometric constant contraction: EMG at (**a**) right forearm, (**b**) left forearm, NIRS at (**c**) right forearm and (**d**) left forearm.

**Figure 9:**
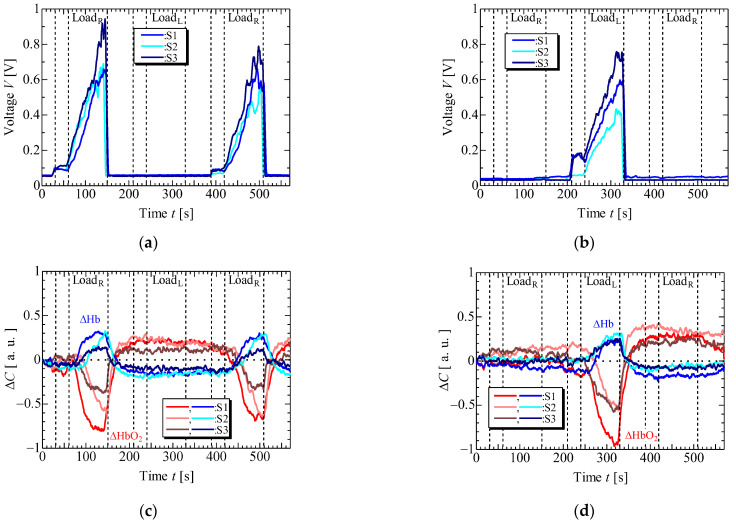
Experimental results of three volunteers in isometric ramp contraction: EMG at (**a**) right forearm, (**b**) left forearm, NIRS at (**c**) right forearm and (**d**) left forearm.

Figure 6 and Figure 7 display the results of EMG and NIRS measurements on the left and right forearms of one volunteer (S1) during isometric constant and ramp contractions, respectively. Figure 8 and Figure 9 present the EMG and NIRS results for three volunteers (S1–S3), averaging data over 5 s intervals per volunteer. These results indicate that during contractions (Figure 6a,b and Figure 8a,b), the measured EMG voltage increased in the forearm where the load was applied, while in ramp contractions (Figure 7a,b and Figure 9a,b), the EMG voltage gradually increased in accordance with the slope of the load. Conversely, EMG voltage remained almost zero in the forearm when the load was not applied and during rest stage. In NIRS measurement, ΔHbO_2_ decreased, and ΔHb increased during the load stage during constant contractions, reaching saturation (Figure 6c,d and Figure 8c,d). In ramp contractions, similar changes occurred during the load stage, nearly reaching saturation. (Figure 7c,d and Figure 9c,d). These values subsequently increased and decreased during the rest period after the loading stage, although this trend was not observed in the EMG measurements. Total-Hb (ΔHbO_2_ + ΔHb) also decreased during the load stage and increased during the rest stage, especially, in constant contraction, where this change was more evident than in ramp contractions. The EMG and NIRS signals were generated by local muscular activity where the sensor was attached.

**Figure 10:**
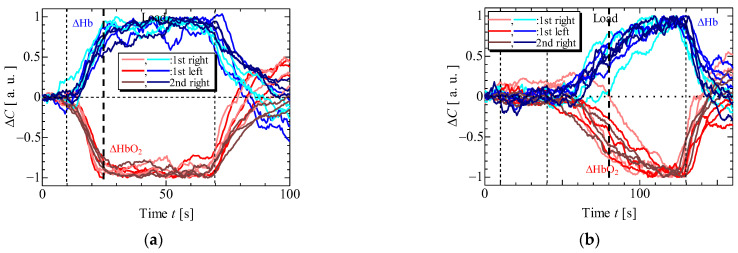
Changes of ΔHbO_2_, ΔHb during the load. (**a**) constant contraction, (**b**) ramp contraction.

In Figure 6a,b and Figure 8a,b, the EMG voltages peaked at approximately 4 ± 4 s (mean ± standard deviation) for three volunteers from the time after the load was applied and gradually decreased during the initial load application to the left and right forearms. For the second load applied to the right forearm, the EMG voltage gradually increased, reaching a maximum value at 34 ± 14 s. Notably, the voltage gradient for the first load differed from that of the second load. While not clearly addressed, this discrepancy might be caused by the adjustments in local muscular activity during the second load, informed by prior experience with the first load. However, such adjustments were not confirmed during the ramp contraction. Therefore, future experiments with multiple volunteers need to be conducted. Figure 10 shows the slopes of ΔHbO_2_, and ΔHb in the left and right forearms when a load was applied. Each slope was obtained by compensating for the initial value with an average of 5 s before the load application. The measured values were then normalized based on the maximum or minimum value during the applied load. The slopes at the first and second loads were almost the same under constant and ramp contractions. To predict muscular fatigue, we estimated that AT would occur at approximately 15 s during constant contraction and approximately 40 s during ramp contraction from the onset of load. This estimation was based on the slopes of ΔHbO_2_ and ΔHb, considering the saturation trend of ΔHb, known as, ΔHb breakpoint, as indicated in previous literature [22,31]. Therefore, we assert that EMG and NIRS measurements at multiple positions effectively evaluate muscular activity, including AT, because the changes in ΔHbO_2_, ΔHb, and EMG exhibit specific characteristics.

However, detection of AT from ΔHb, or ratio of ΔHb and EMG signal during the ramp contraction remains challenging due to insufficient measurement accuracy, necessitating improvements in measurement accuracy. Furthermore, we must compare AT estimates obtained via the sensor during local muscular activity with those from respiration gas sensors.

## 5. Conclusions

Development of sensors capable of measuring several local muscular activities and predicting the fatigue of local muscles is vital to prevent sports-related injuries and damage. We have successfully designed a wireless 2-channel layered sensor system, enabling simultaneous EMG and NIRS measurements during constant isometric and ramp contractions in both forearms to evaluate the relationship between the EMG and NIRS signals and local muscular activity. Our results demonstrate that layered sensors are effective in measuring local muscular activity simultaneously using EMG and NIRS. The EMG and NIRS signals were generated as a result of local muscular activity when sensors were applied. The changes in EMG signals were significantly different than those of NIRS (the ΔHbO_2_, ΔHb, and Total-Hb) in the constant and ramp exercises. Moreover, NIRS signals exhibited post-activity changes not observed in EMG signals. It has been estimated that AT in local muscles can be detected based on changes in ΔHb and ΔHbO_2_. Therefore, the layered sensor system holds potential for a more detailed analysis of multiple local muscular activities, including AT detection.

In future studies the layered sensor will be useful for analyzing several local muscular activities, such as lateral and medial vastus muscles, upper and forearm muscles, and evaluating fatigue in their muscular activities, such as differences in AT estimates between EMG and NIRS for each muscle, and explore the relationship between EMG and NIRS signals. In this sensing system, calculating the mean power frequency as part of EMG frequency analysis was not feasible. A processing system capable of frequency analysis will be developed. Therefore, AT prediction is possible using a layered sensor because the mean power frequency of the EMG signal is related to the prediction of AT. Furthermore, we aim to develop wearable sensors and signal-processing devices. The layered sensor system holds promise for application in sports science and rehabilitation, including prediction of injury and accidents in sports, and effective recovery of muscular activity.

## Data Availability

The data presented in this study are available on request from the corresponding author.
